# Increased antitumor efficacy of ginsenoside Rh_2_ via mixed micelles: *in vivo* and *in vitro* evaluation

**DOI:** 10.1080/10717544.2020.1825542

**Published:** 2020-09-30

**Authors:** Xiaojing Xia, Jin Tao, Zhuwa Ji, Chencheng Long, Ying Hu, Zhiying Zhao

**Affiliations:** a Department of Pharmaceutics, Zhejiang Pharmaceutical College, Ningbo, PR China; b Department of Traditional Chinese Medicine, China Pharmaceutical University, Nanjing, PR China

**Keywords:** Ginsenoside Rh2, mixed micelles, Solutol^®^ HS15, TPGS, A549 cell, antitumor

## Abstract

The aim of this work is to apply Solutol^®^ HS15 and TPGS to prepare self-assembled micelles loading with ginsenoside Rh_2_ to increase the solubility of ginsenoside Rh_2_, hence, improving the antitumor efficacy. Ginsenoside Rh_2_-mixed micelles (Rh_2_-M) were prepared by thin film dispersion method. The optimal Rh_2_-M was characterized by particle size, morphology, and drug encapsulation efficiency. The enhancement of *in vivo* anti-tumor efficacy of Rh_2_-M was evaluated by nude mice bearing tumor model. The solubility of Rh_2_ in self-assembled micelles was increased approximately 150-folds compared to free Rh_2_. *In vitro* results demonstrated that the particle size of Rh_2_-M is 74.72 ± 2.63 nm(PDI = 0.147 ± 0.15), and the morphology of Rh_2_-M is spherical or spheroid, and the EE% and LE% are 95.27 ± 1.26% and 7.68 ± 1.34%, respectively. The results of *in vitro* cell uptake and *in vivo* imaging showed that Rh_2_-M could not only increase the cell uptake of drugs, but also transport drug to tumor sites, prolonging the retention time. *In vitro* cytotoxicity and *in vivo* antitumor results showed that the anti-tumor effect of Rh_2_ can be effectively improved by Rh_2_-M. Therefore, Solutol^®^ HS15 and TPGS could be used to entrapping Rh_2_ into micelles, enhancing solubility and antitumor efficacy.

## Introduction

Ginsenoside Rh_2_ (Rh_2_) is a natural active component purified from *Rhizoma Ginseng*, which has many pharmacological activities, such as enhancing immunity (Lee et al., 2018), delaying aging (Chu et al., 2014), relaxing blood vessels, improving the insufficiency of cardiovascular and cerebrovascular blood supply (Lo et al., 2017) and anti-tumor efficiency (Chen & Qiu, 2015). Among them, the antitumor effect of Rh_2_ has been an attractive topic recently. Studies show that Rh_2_ can inhibit melanoma (Wang et al., 2017), glioma (Kim et al., 2007), liver cancer (Yang et al., 2016), breast cancer (Zare-Zardini et al., 2018), and lung cancer (An et al., 2013). Rh2 inhibits tumor tissue growth by inhibiting the proliferation of tumor cells, inducing apoptosis (Zhuang et al., 2018), inhibiting tumor invasion (Li et al., 2015), migration and angiogenesis (Chen et al., 2018), and reversing/delaying multidrug resistance (Wen et al., 2015). The growth of tumor cells was interfered by regulating tumor microenvironment. Although the promising antitumor properties of Rh_2_, there is a fundamental challenge for the application of Rh_2_, extremely low solubility and rapid plasma elimination (Gu et al., 2009; Li et al., 2017). A few studies have reported that some drug delivery systems could enhance increase hydrophilicity and blood circulation of insoluble drugs by using a mixed micelles prepared with amphiphilic surfactants, and the possible drug loading and antitumor effect still need to be improved (Chen et al., 2014; Jin et al., 2016).

Solutol ^®^HS15 is a kind of nonionic surfactants with high safety, biocompatibility and commercial availability (Hou et al., 2016). It was originally developed as a parenteral formulation. For example, Solutol^®^HS15 was clinically developed as an osmotic enhancer for nasal spray (Williams et al., 2018). Recently, it is often used to improve the aqueous solubility of drug of Biopharmaceutical Classification System (BCS) ClassII (Patel et al., 2011). However, studies on Solutol^®^HS15 are still limited, especially when used as a nanocarrier. It has been found that Solutol^®^HS15 can be assembled on lecithin to form lipid micelles to increase the solubility of insoluble drugs (Shaji & Varkey, 2016). TPGS is prepared by the esterification of vitamin E succinate with the hydroxyl of polyethylene glycol (PEG) 1000, which is FDA certified surfactant of polymer materials. And TPGS has many beneficial characteristics of high encapsulation efficiency and long internal circulation time. Besides, TPGS which forming a block copolymer micelle had the ability to inhibit the activity of P-gp (Dintaman & Silverman, 1999; Collnot et al., 2007; Choudhury et al., 2017; Jin et al., 2017; Liu et al., 2018). P-gp is one of the important proteins encoded by MDR1 gene and promotes the multidrug resistance of tumor cells. The main function of P-gp is to pump drugs out of cells, thereby limiting the injuring effect of drugs on tumor cells. TPGS binds to non-transporter active sites on P-gp, resulting in a change in the conformation of P-gp, and then lose the transshipment function of P-gp. Finally, the pump out of the drug by P-gp was inhibited, and the dosage of the needed drug was reduced, which resulted in higher tumor inhibition effect. Meanwhile it has been reported that based on the theory of synergistic effect, self-assembled micelles composed of two or more polymers exhibit preferable characteristics to micelles formed from only one surfactant (Hou et al., 2016).

Hence, in this paper, Solutol ^®^HS15/TPGS-mixed micelles was prepared to increase the solubility and retention time of tumor site of Rh_2_, thus, enhancing the antitumor effect of Rh_2_. The mixed micelles containing Rh_2_ were prepared by the thin-film dispersion method. The particle size, Zeta potential, and morphology were characterized by Malvern system particle size analyzer and transmission electron microscope, respectively. The *in vitro* release behavior of Rh_2_-mixed micelles（Rh_2_-M） was detected by dialysis method. MTT assay was used to detect the cytotoxicity of Rh_2_-M *in vitro* and its effect on cell migration, invasion and apoptosis were examined. *In vivo* imaging was used to visualize the enrichment ability and retention time of Rh_2_-M in tumor target. The tumor-bearing nude mice were used to evaluate the *in vivo* anti-tumor effect of Rh_2_-M.

## Materials and methods

### Materials

Ginsenoside Rh_2_ of >98% purity was purchased from Nanjing Jingzhu Biotechnology, Jiangsu, China. Solutol^®^ HS15 was purchased from BASF, Shanghai, China. TPGS was purchased from Aladdin, China. Methyl thiazolyl tetrazolium (MTT) and dimethyl sulfoxide (DMSO) were purchased from Nanjing Sunshine Biotechnology, Jiangsu, China. Coumarin-6 and DAPI were purchased from Sigma, Shanghai, China. DiR iodide (DiR) and the human lung adenocarcinoma cell line A549 was purchased from Nanjing KeyGen Biotech, Jiangsu, China. All reagents were of analytical grade except methanol, which was of chromatographic grade.

### Animals

Male athymic nude mice（22 ± 2g, SPF）were obtained from Changzhou Cavens Lab Animal Co., Ltd. (Changzhou, China, SCXK2011-0003). All mice were given distilled water and kept under pathogen-free conditions (25 ± 0.5 °C) for 3 days to acclimatize to the experimental environment. All animal experimental procedures were approved by the Institutional Animal Care and Use Committee (IACUC), Jiangsu Provincial Academy of Chinese Medicine’s Experimental Animal Center. Prior to the tests, animals were fasted for 12 h and provided with only water.

### Preparation of drug-loaded micelles

The Solutol^®^ HS15-TPGS-mixed micelles was prepared using the film dispersion method. In the preparation of Rh_2_-loaded mixed micelles, 17.5 mg Rh_2_, 140 mg Solutol^®^ HS15 and 60 mg TPGS were dissolved in absolute ethyl alcohol (the ratio of Solutol^®^ HS15 and TPGS is 7:3). Subsequently, volatile organic solvent was evaporated to dry in a rotary evaporator under 60 °C for 10 min, and dry film formed in the eggplant-shaped flask. 5 mL of Distilled water was added, and the solution was filtered with 0.45 μm filter membrane to remove the unencapsulated Rh_2_.

The preparation of coumarin-6-mixed micelles (C6-M) and DiR-mixed micelles (DiR-M) is the same as above method.

### Characterization of drug-loaded micelles

The zeta potential, hydrodynamic diameter and particle size distribution of the Rh_2_-M were measured by Malvern system (ZEN-3600, Malvern Instruments, Worcestershire, UK). Micellar solutions were filtered with a 0.45 µm filter prior to the measurement. All the values were the average of at least three parallel measurements.

Transmission electron microscopy (TEM) pictures of the copolymer micelles were captured on a transmission electron microscopy (TEM; JEM-200CX, JEOL, Tokyo, Japan). Briefly, A drop of Rh_2_ micelles solution (1.0 mg/mL) was dipped into the copper grid, mixed with a drop of 2% (w/v) phosphotungstic acid. After the deposition, samples were observed under TEM.

The drug-loading efficiency (LE%) and encapsulation efficiency (EE%) were determined using a 4.6 mm × 250 mm Hypersil ODS C18 5.0 μm column (Thermo, Waltham, MA) by HPLC (Agilent 1260, Palo Alto, CA). Briefly, the Rh_2_-M solution was diluted with methanol to dissociate the micellar nanoparticles and centrifugation at 15,000 rpm for 15 min. Then the supernatant was filtrated by 0.45 μm filter membrane and the concentration of Rh_2_ was determined by HPLC. The composition of the mobile phase was acetonitrile: water: phosphoric acid (65:35:0.2, v/v/v) with a flow rate of 1.0 mL/min at 35 °C. Quantitation was achieved using UV detection at 203 nm. A typical injection was 20 μL. The method was linear over a wide concentration range of 0.02–1.0 mg/mL. Assay method had been verified by methodology.

Then, use the following formula to calculate the LE% and EE%:

$$\mathrm{EE}\%=\frac{\text{weight of }{Rh}_{2}\text{ in micelles}}{\text{weight of the initial }{Rh}_{2}}\times 100\%$$

$$\mathrm{LE}\%=\frac{\text{weight of }{Rh}_{2}\text{ in micelles }}{\text{weight of micelles containing }{Rh}_{2}\;}\times 100\%$$

### *In vitro* release study

The *in vitro* release from the copolymer micelles Rh_2_-M were inspected using dialysis method (Wang et al., 2019). A phosphate buffer solution (PBS, pH = 7.4) including 0.5% Tween 80, which was chosen as a dissolution medium under suitable sink conditions. 1.0 mL of free Rh_2_ and aliquots of Rh_2_ incorporated micelles (3.5 mg/mL of Rh_2_) were transferred into the dialysis bags (molecular weight cutoff size 3,500 Da), which were tightened and put into 100 mL dissolution medium. Then concussion in a thermostatic oscillator (37 ± 0.5 °C, 100 rpm), 1 mL dissolution medium was taken out in predetermined interval(0, 0.25, 0.5, 1, 4, 8, 12, 24, and 48 h), and replaced with an equal volume of fresh medium. The concentration of Rh_2_ was measured by HPLC and *in vitro* Rh_2_ release profiles were plotted with cumulative drug release as a function of time. Experiments were conducted in triplicate.

### *In vitro* cytotoxicity analysis

Inhibitory effect of Rh2 and Rh2-M on proliferation of A549 cells was detected by MTT assay (Quandt et al., 2014). In logarithmic growth phase, A549 cells were digested with trypsin. Single cell suspension was obtained from 10% fetal bovine serum DMEM. The cells were diluted to 4 × 10 ^4^/mL and inoculated into 96-well cell culture plate with 100 μ L culture medium in each hole. PBS was added to the bottom and both sides of the cells. A549 cells were cultured at 37 °C in a 5% CO_2_ atmosphere for 24 h, subsequently, free Rh_2_ and Rh_2_-M diluted with different concentrations were added into the culture medium. After 24 h of incubation, the culture medium was removed and added with 100 μL MTT solution (0.5 mg/mL) and incubated for 4 h. MTT was discarded and then dimethyl sulfoxide solution (DMSO, 100 μL) was added. The medium was shaken at a speed of 50 rpm for 10 min. The absorbance of the sample was measured at 570 nm using the microplate reader (Bio-Rad Laboratories, Hercules, CA), and the IC_50_ was calculated. Six parallel composite holes were set for each concentration:

$$\mathrm{Cell}\;\mathrm{viability}=\frac{A_{\mathrm{sample}\;}-A_{\mathrm{blank}}}{A_{\mathrm{control}\;}-A_{\mathrm{blank}}}\times 100\%$$

### Cellular uptake

Courmanrin-6 was used as a model Active pharmaceutical ingredient for cellular uptake (Rivolta et al., 2011). A549 cells in logarithmic growth phase were inoculated with a density of 1 × 10^5^/well on a 24 well culture plate, incubated for 24 h to make A549 cells adhere to the wall. After the old culture medium was abandoned, coumarin-6 and C6-M were added respectively and cultured for 4 h. Subsequently, cold PBS was added to terminate cell uptake and the cells were flushed by PBS for 3 times. Immobilized cells at 4 °C for 30 min with anhydrous ethanol and stained with DAPI(5 μg/mL). The cells were incubated for 10 min and washed with PBS for 2 times (Yang et al., 2017). The uptake of cells was observed under fluorescence microscope.

### Cell migration

Scratch assay was widely available format to study cell migration (Hulkower & Herber, 2011). The cells in logarithmic growth period were digested and inoculated into the six-hole plate. when the cell aggregation was about 80%, the sterile gun head was used to draw the line evenly in the six-hole plate. After the floating cells were washed off with PBS, the fresh culture medium containing free Rh_2_ (26.48 μg/mL of Rh_2_) and Rh_2-_M (26.48 μg/mL of Rh_2_) were replaced and cultured in a cell incubator. After 24 h of incubation, the cells were taken out and photographed (magnification multiple 100), the cell migration distance was measured.

### *In vivo* imaging

The nude mice of the same weight were inoculated A549 cells on their backs. After the tumor grew to 60 mm, DiR fluorescent dye(0.5 mg/mL of DiR) and DiR-mixed micelles (0.5 mg/mL of DiR) were injected into the tail vein, respectively (Jiang et al., 2012). The nude mice were anesthetized at 0.5 h, 2 h, 4 h, 8 h, 12 h, and 24 h, and the fluorescence intensity of tumor sites and organs *in vivo* were detected on an *in vivo* imaging system (NightOWLII LB983; Berthold, Wildbad, Germany). After 24 h, the nude mice were euthanized, planed and the heart, liver, spleen, lung, kidney, and tumor were collected to detect fluorescent expression on the living imager.

### *In vivo* antitumor activity

The logarithmic A549 cells were inoculated on the back of nude mice (22 ± 2 g). When the tumor reached about 50 mm, the nude mice were randomly grouped. Nude mice were randomly allocated to model group (0.2 mL of normal saline), positive drug group (2 mg/kg of cisplatin), Rh _2_ group (15 mg/kg of Rh_2_), and Rh_2_-M group (15 mg/kg of Rh _2_). Tail vein injections were administered for 18 days, and dosing five times (Yang et al., 2016). Weight and tumor size measured before each dose. The nude mice were euthanized on the third day after the last administration, and the tumor was taken and weighed and photographed. The tumor volume (V) is calculated as: *V* = *a* × *b*^2^/2, where *a* and *b* are the length and width of the tumor, respectively. According to the above measurements, the relative tumor volume was calculated, and antitumor activity was evaluated by the relative tumor growth rate (*T*/*C*):

$$\mathrm{RTV}\;=\;V_{t}/V_{0}$$

$$T/C\;=\;T_{\mathrm{RTV}}/C_{\mathrm{RTV}}\times 100\%$$

where *V*_0_ is the tumor size at 0 day; *V*_t_ is the tumor volume at each measurement; *T*_RTV_ is the RTV of the treatment groups; and *C*_RTV_ is the RTV of the control group.

The tumor and the liver of each group were kept in 10% formaldehyde bag. The tissue was then embedded in a wax block for easy slicing. After two xylene and gradient alcohol (100%, 90%, 80%, and 70%) dew axing and hydration, the cut wax sheet is dyed in sequence by using wood essence and eosin, and finally the wax sheet is sealed with a neutral gum. Tissue samples were observed and photographed microscope.

### Histological studies

The samples of and livers in studies were removed from mice and post fixed in 4% neutral formaldehyde, then embedded in paraffin wax for pathological cut sheet. Sections were stained with hematoxylin and eosin and observed with an upright microscope (Eclipse Ni-U, Nikon, Japan).

### Statistical analysis

All data are represented by mean ± SD, and SPSS version 19.0 (SPSS Inc., Chicago, IL) was used to detect the significance between groups (*p* < .05). Differences between two groups were assessed using Student’s *t*-test.

## Results

### Mixed micelle formulation and characterization

**Figure 1.**
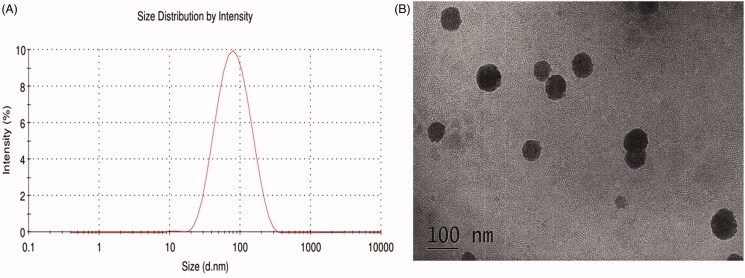
Size distribution and TEM of Rh_2_-mixed micelles.

The Rh_2_-mixed micelle solution prepared by thin-film dispersion method was clarified without obvious flocculent precipitation and had light blue emulsion light. The optimized formulation is Solutol^®^ HS15: TPGS = 7: 3, and the LE% and EE% are 7.68 ± 1.34% and 95.27 ± 1.26%, respectively. The concentration of Rh_2_ in Rh_2_-M is 3.33 ± 0.04 mg/mL, which is an approximately 150 time higher than that of Rh_2_ in water(23.1 ± 1.5 μg/mL) (Souza et al., 2019). As seen in Figure 1, the Rh_2_-M exhibited spherical or spheroid. The particle size of the prepared micelles was uniform (PDI = 0.147 ± 0.15), the average particle size was 74.72 ± 2.63 nm (Figure 1(A)). The ζ potential of the Rh_2_-M was –(26.2 ± 0.9)mV, which indicated the high ability to hinder the probability of coalescence and thereby maintaining the homogeneity of nanodroplets (Honary & Zahir, 2013). Therefore, Rh_2_-M is prepared successfully with almost spherical and uniform shapes and increased the solubility.

### *In vitro* release of Rh_2_-loaded mixed micelles

**Figure 2.**
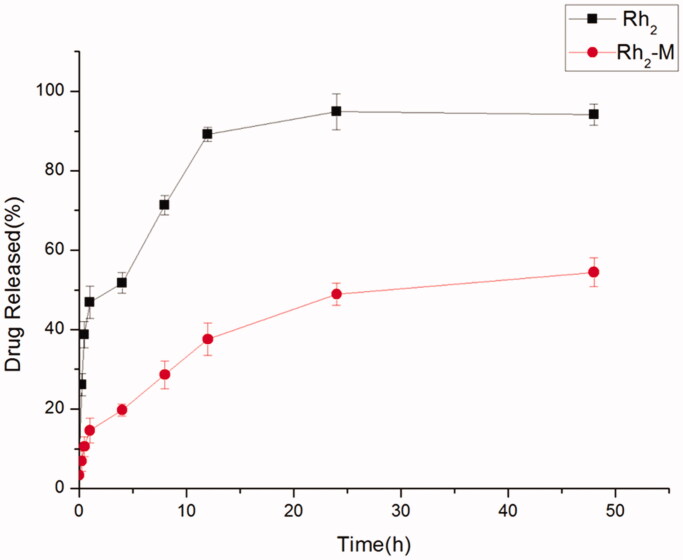
*In vitro* release profiles of Rh_2_-M and free drug, data represent the mean ± SD (*n* = 6).

The slow release of drug by sustained-release preparations is helpful to retard the absorption rate of drug into the body, thus, making the drug play a better therapeutic effect. A sudden release occurs at the initial stage of Rh_2_-M release, followed by a sustained and slow release of the drug, and sustained release of the Rh_2_-M within 48 h. Rh_2_ has released 51.73% in 4 h, and completely released in 24 h (Figure 2), demonstrating that Rh_2_-M has a certain slow-release effect, which may enhance the antitumor efficacy of Rh_2_.

### *In vitro* cytotoxicity of micelles

**Figure 3.**
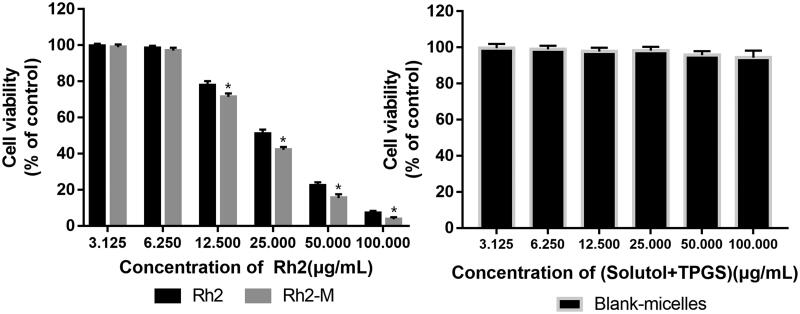
Cell cytotoxicity in A549 Cells. *Note*: data shown represent the mean values of six experiments ± SD. *p<.05.

As shown in Figure 3, Rh_2_ and Rh_2_-M showed cytotoxicity against A549 cell line, and the latter exhibited higher cell cytotoxicity. Respectively, the results showed that the IC_50_ of Rh_2_ and Rh_2_-M were 26.48 ± 2.13 μg/mL and 21.71 ± 1.85 μg/mL, which suggest that Rh_2_-M can enhance the inhibitory effect of Rh_2_ on lung cancer cells (*p* < .05). The IC_50_ of blank micelles was greater than 100 μg/mL. It was inferred that the nanocarrier had good biocompatibility.

### Cellular uptake

**Figure 4.**
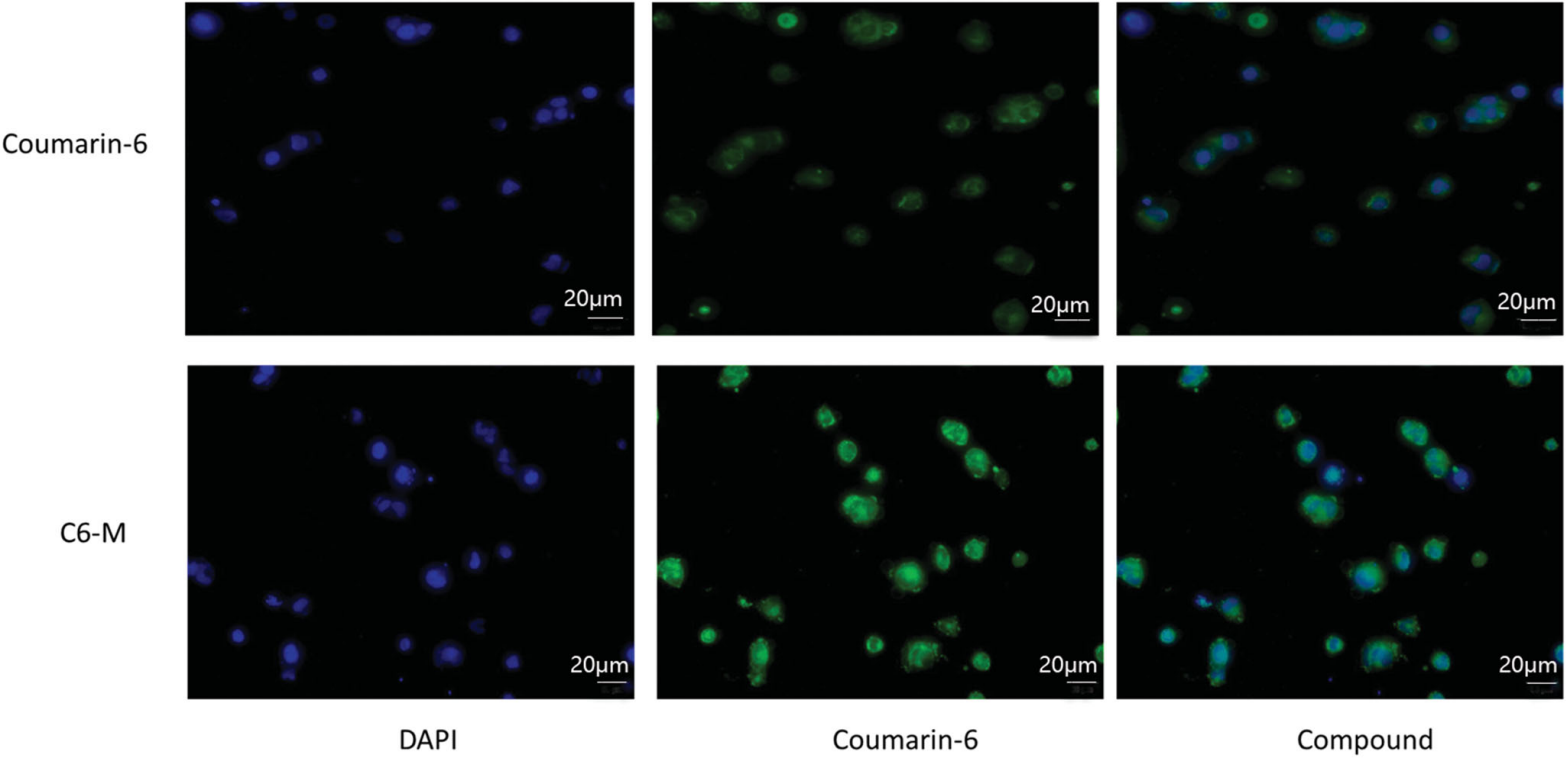
Cell uptake of coumarin-6 (C6) and C6-M by A549 cells observed by fluorescence inverted microscope.

The fluorescent dye coumarin-6 (C6) is often used to replace insoluble drugs in order to investigate whether nano pharmaceuticals can improve the efflux of drugs. In this paper, coumarin-6 was coated with mixed micelles composed of Solutol^®^ HS15 and TPGS (C6-M), and then A549 cells was cultured in serum-free medium containing C6 and C6-M for 4 h. As depicted in Figure 4, green fluorescence represents C-6, blue fluorescence represents nucleus. At the same time, the fluorescence intensity of C6-M can be used as preliminary judgments of the ability of cell uptake of drugs. Four hours later, the green fluorescence intensity of C6-M was significantly stronger than that of C6, indicating that the mixed micelles with Solutol^®^ HS15 and TPGS could enhance the cell uptake of drugs. The possible reasons were the small size of nanoparticle and the amphiphilic carrier, in addition to this, TPGS might inhibit the activity of P-gp and then the cellular uptake to C6 was improved significantly.

### Inhibitory effect of Rh_2_-M on cell migration

**Figure 5.**
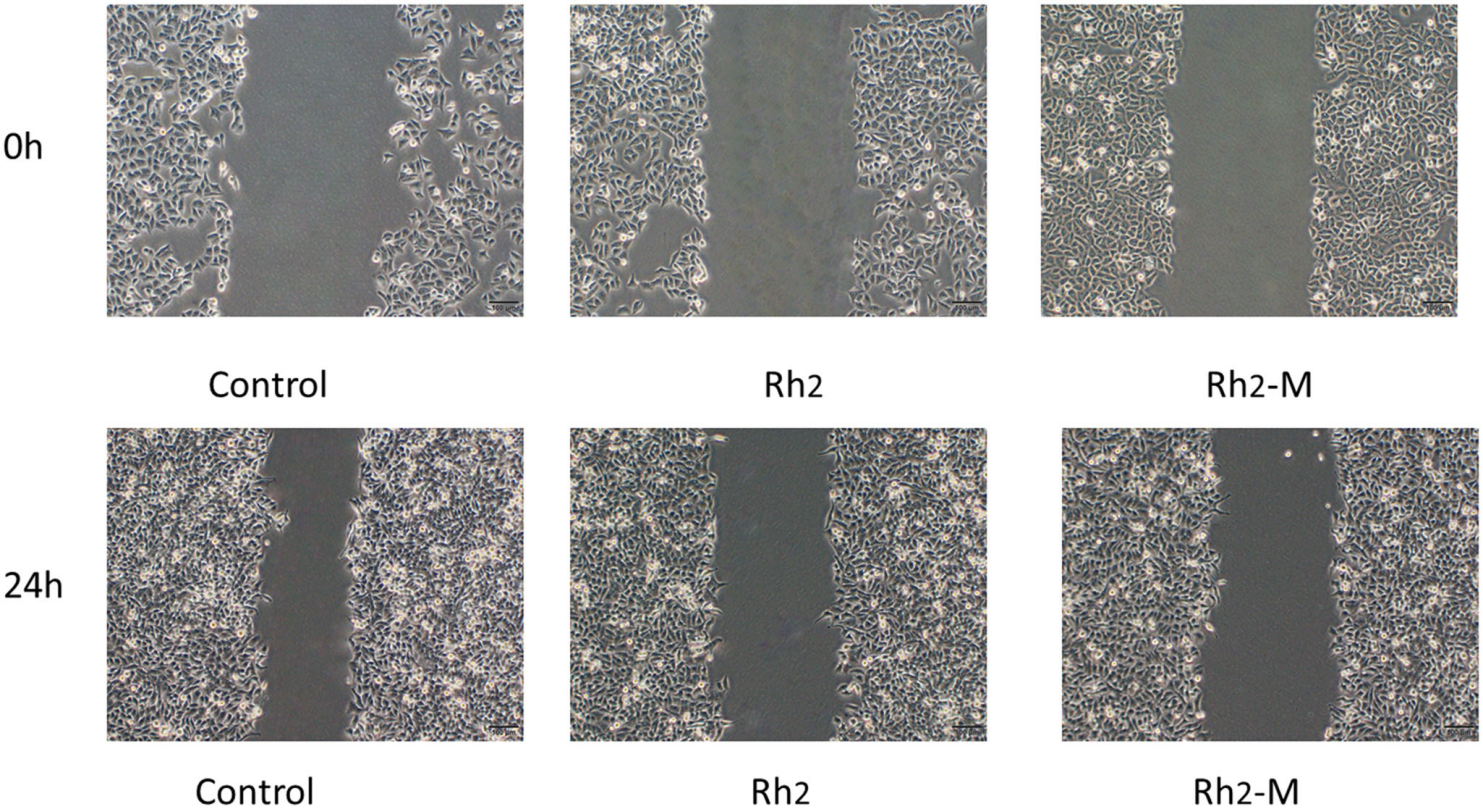
Representative micrographs pictures of A549 scratch assays at 0 h and 24 h.

The migration of tumor cells is the process that tumor cells invade lymphatic vessels, blood vessels, or body cavities from their primary site and the tumor cells continue to grow, forming a tumor as the primary tumor. Inhibition of tumor cell migration via drugs can effectively prevent tumor deterioration, which is one of the important indexes to evaluate the efficacy of antitumor drugs. Compared to blank control, the migration length of A549 cells was shortened after Rh_2_ -M (159.77 ± 3.80 μm) and Rh_2_ (109.25 ± 4.84 μm, *p <* .05) treatment, respectively (Figure 5).

### *In vivo* imaging

**Figure 6.**
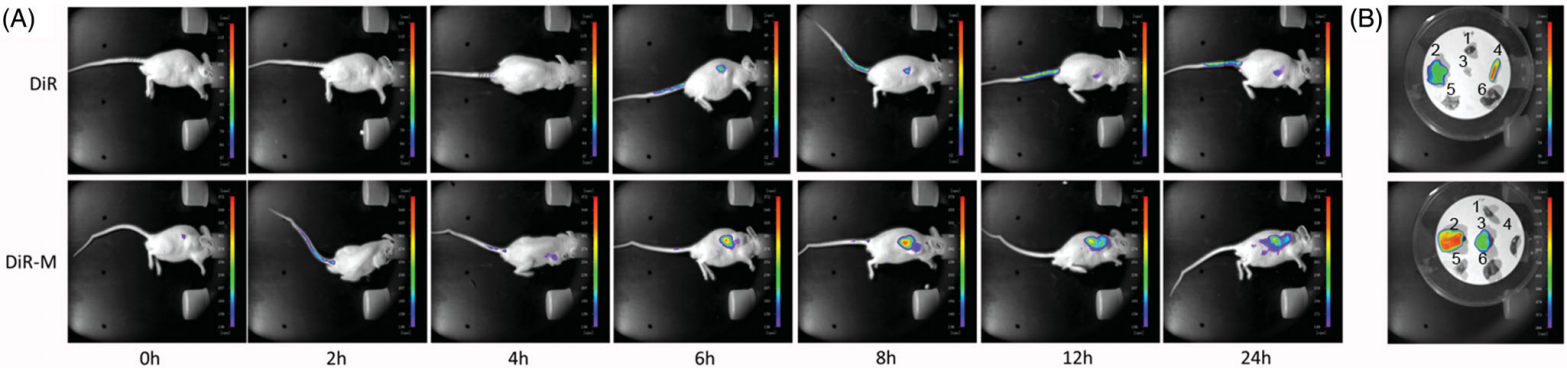
Confocal laser scanning microscopy (CLSM)of (A) tumor-bearing nude mice after administration of free DiR and DiR mixed micelles at 0, 2, 4, 6, 8, 12, and 24 h and (B) mice main organs after 24 h. (1) Heart, (2) liver, (3) tumor, (4) spleen, (5) lung, and (6) kidney.

As shown in Figure 6, the fluorescence expression of DiR dye group is mainly concentrated in the liver. In the DiR-M group, the fluorescence was displayed at the tumor site at 6 h and showed an increasing tendency with the time lapsed. After 24 h of euthanasia, the nude mice were euthanized and the heart, liver, spleen, lung, kidney, and tumor were taken to take pictures. The results showed that there was still fluorescence expression in the tumor site of DiR-M group 24 h later. These results indicated that Rh_2_-M can increase the concentration and retention time of Rh_2_ in tumor site, thus enhancing the tumor inhibition effect of Rh_2_ *in vivo*.

### *In vivo* antitumor activity

**Figure 7.**
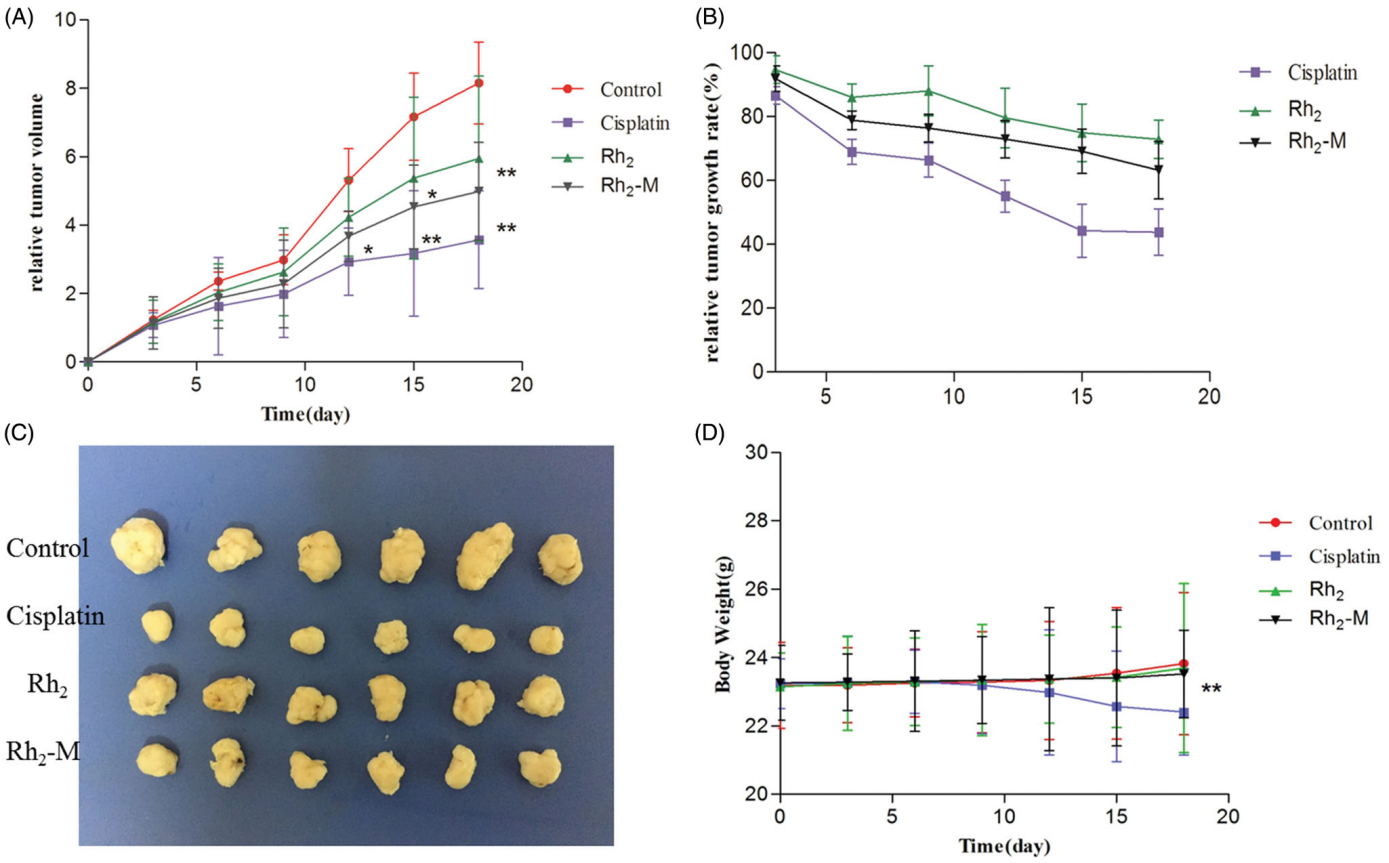
Effect on the inhibition of tumor growth of cisplatin, Rh_2_, Rh_2_-M and control group in tumor-bearing nude mice. Data represent the mean ± SD (*n* = 6). (A) Relative tumor volume; (B) relative tumor growth rate; (C) image of tumor; (D) body weight.

The *in vivo* antitumor effect of Rh_2_-M *via* nude mice bearing tumor model was investigated (Figure 7). The tumor volume in the positive group was significantly lower than that in the control group during the third administration (*p* < .05). At the same time, the body weight of the positive group decreased gradually with the increase of administration times. It shows that cisplatin has a significant inhibitory effect on lung cancer, but with serious side effects on the body weight. Compared with the model group, Rh_2_ and Rh_2_-M have inhibitory effect on the growth of lung cancer tumor, and with the prolongation of administration time, the inhibitory effect is enhanced. After the fifth dose, the tumor volume of Rh_2_-M group was significantly lower than that of model group (*p <* .05). The weight of nude mice in Rh_2_ and Rh_2_-M groups was not significantly different from that in model group, indicating the safety of Rh_2_ and Rh_2_-M.

At the end of the experiment, the nude mice were planned, and the tumors were weighed. Results as shown in the table, the tumor weight of Rh_2_-M was significantly lower than that of the model group. Therefore, the above results indicate that Rh_2_ has a certain inhibitory effect on the growth of lung cancer, and Rh_2_-M can enhance the inhibitory effect of Rh_2_ on lung cancer.

### Histological studies

**Figure 8.**
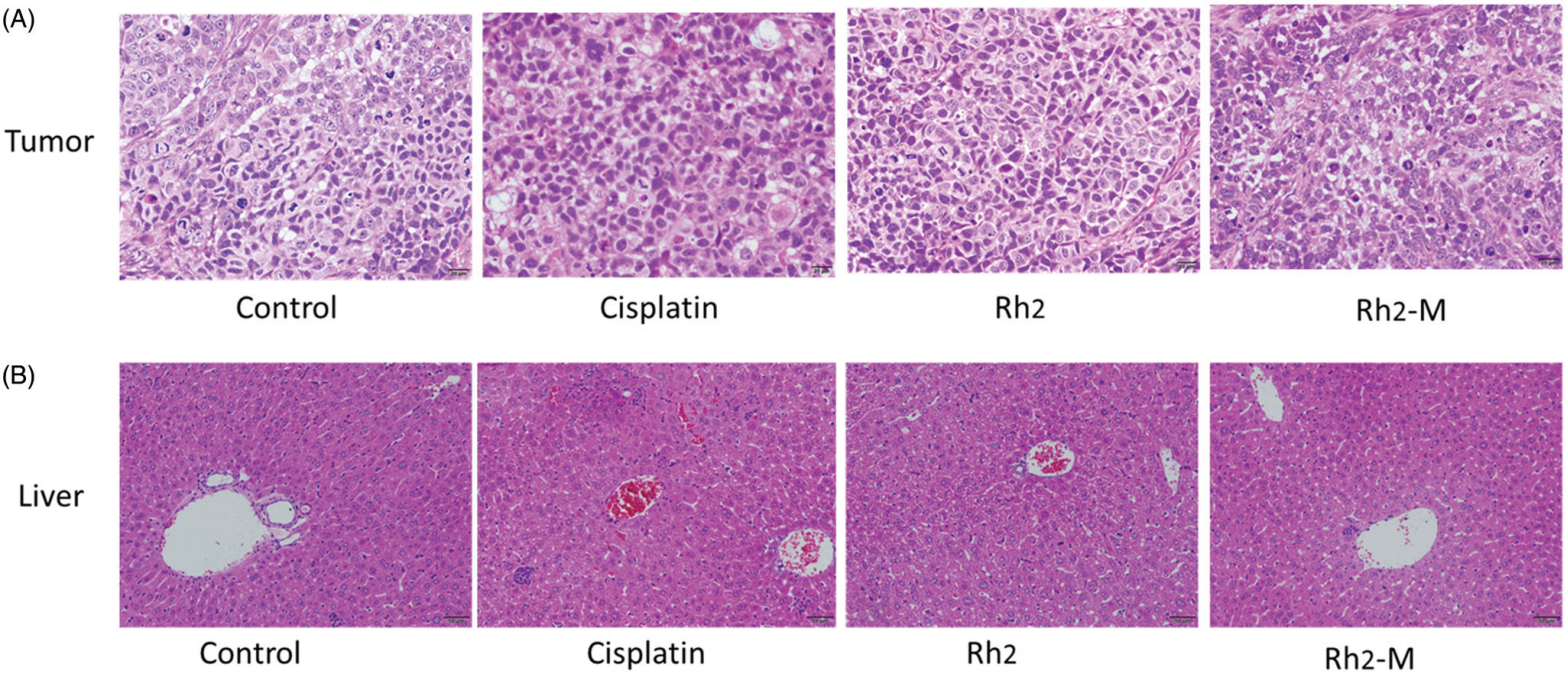
Histopathological section of the tumor and liver treated with saline solution, cisplatin, Rh_2_, and Rh_2_-M.

The results of hematoxylin–eosin (HE) staining showed that the tumor cells in the positive group and the Rh_2_-M group had different degrees of necrosis, while the tumor cells in the positive group and the Rh_2_-M group had slight apoptosis, which further indicated that Rh_2_-M had inhibitory effect on lung cancer cells (Figure 8).

The liver is the main metabolic organ of the body. Drugs must enter the systemic circulation through the liver before distributed to the diseased site. Therefore, the liver is greatly affected by the side effects of drugs. The toxic and side effects of anti-tumor drugs and new nano-preparations on liver should also be included in the safety evaluation. The results of Figure 8 showed that there were multiple focal necroses (moderate necrosis) in the liver tissue of the positive group, and the hepatocytes disappeared in the necrotic area. There was no obvious hepatic lesion in Rh_2_ and Rh_2_-M groups.

## Discussion

Rh_2_ is one of the most effective monomers in ginsenosides. However, due to the poor solubility of Rh_2_, its clinical application is restricted. In recent years, researchers have developed a variety of nano pharmaceuticals to improve the solubility of Rh_2_, such as self-microemulsions (Yang et al., 2017), nanoparticles (Singh et al., 2017; Kim et al., 2019), liposome-based delivery systems (Yang et al., 2016), and pH-sensitive mixed micelles (Zhuang et al., 2018).

Polymer micelles can be used as carriers of antineoplastic drugs to effectively improve drug defects. The polymer carriers spontaneously form micelles with a hydrophobic core and a hydrophilic shell in water. Hydrophobic drugs are encapsulated into the core and delivered to tumor targets, while reducing accumulation in other areas and protecting the drug from inactivation in biological media, thus the antineoplastic effect of the drug is improved. In this paper, Rh_2_-mixed micelles were successfully prepared by the thin film dispersion method. The size of the prepared Rh_2_-M particles is uniform; the morphology is spherical or spherical, and the average particle size is 74.72 ± 2.63 nm. Encapsulation efficiency and drug loading efficiency of Rh_2_-M were 95.27 ± 1.26% and 7.68 ± 1.34%, respectively. The results of *in vitro* release showed that Rh_2_-M was released slowly within 48 h, and the accumulative release percentage was 54.42 ± 3.21%. Therefore, the prepared Rh_2_-M can effectively increase the solubility of Rh_2_ and has a certain slow-release effect, which lays a certain foundation for increasing the anti-tumor effect of Rh_2_.

The hemolysis test, stability in physiological conditions, and shelf stability were evaluated further. The results of hemolysis test (Song et al., 2014) showed that the hemolysis percentage of Solutol:TPGS (7:3) was 0.73% (0.5 mg/mL of mix nanocarrier), while the hemolysis percentage of Rh_2_-M (0.1 mg/mL of Rh_2_) was 1.56% (*n* = 3). The Rh_2_-loaded Solutol TPGS mix micelles were mixed with appropriate volumes containing 10%vol% fetal bovine serum (Zhang et al., 2014). The diluted samples were incubated at 37 °C. Samples were taken to determine the particle size at 2, 4, 6, 8, 12 h, and 24 h. The experiments were repeated three times, and the average values are reported(*n* = 3).There was a little increasing in particle size (78.91 ± 2.15 nm after storage). And 93.3 ± 1.6% of the Rh_2_ content in the mix micelles remained. Rh_2_-M could be preserved for more than 21 days at 4° C and 5–6 days at 25 °C. It can be inferred that the mix micelles have less hemolysis, good biocompatibility, and Rh_2_-M are stable in physiological conditions.

In the further study, MTT cell proliferation assay and cell migration test were used to evaluate the cytotoxicity of Rh_2_ and check whether Rh_2_-M could increase the effect. The results showed that the IC_50_ of Rh_2_-M was 21.71 ± 1.85 μg/mL, which was superior to that of IC_50_ of Rh_2_ (26.48 ± 2.13 μg/mL). And Rh_2_-M could enhance the inhibitory effect of Rh_2_ on the migration of lung cancer A549 cells. And the blank Solutol^®^HS15 and TPGS self-assembled micelles have no obvious cytotoxicity to A549 cells. The results suggest that Rh_2_-M enhances the proliferation of A549 cells by Rh_2_. Further studies have found that Rh_2_ has a good inhibitory effect on tumor cell migration, and Rh_2_-M can enhance this inhibitory effect.

It has been found that TPGS can inhibit drug delivery mediated by P-gp and inhibit multidrug resistance (MDR) (Gao et al., 2016). As a result, the concentration of the drugs entered the cell is increased, and the antitumor effect of the drugs increases. On the other hand, it was found that micelles with particle size below 200 nm could avoid being swallowed by reticuloendothelial system and make more drugs enter the target site (Lai et al., 2010). The results of *in vivo* imaging showed that the fluorescence expression of DiR dye encapsulated by micelles of Solutol^®^HS15 and TPGS was much higher than that of free DiR dye, and the retention time of DiR-M in tumor site was more than 24 h.

The increase of cell uptake and drug concentration in tumor site directly leads to the increase of antitumor effect of Rh_2_. Tumors bearing nude mice were selected to evaluate the antitumor effect *in vivo*. The statistical results showed that the tumor inhibition rate of Rh_2_-M was 38.84 ± 5.92%, which was significantly higher than that of the model group (*p* < .05). Rh_2_-M had no obvious inhibitory effect on the body weight of nude mice and no obvious toxicity to the liver, while the weight loss of the positive group was obvious. Herein, the prepared Rh_2_-M can enhance the anti-tumor effect of Rh_2_ and has no significant toxicity to the body, thus it is a potential and safe anti-tumor nanometer preparation.

## Conclusion

The solubility and the antitumor effect of Rh_2_ were enhanced by Rh_2_-M using Solutol^®^HS15 and TPGS. The LE% and multidrug resistance of Rh_2_ could be increased by Rh_2_-M. Meanwhile, Rh_2_ was released in a sustained behavior from Rh_2_-M, and Rh_2_-M could directly increase Rh_2_ uptake and tumor site concentration. The experimental results of anti-tumor effect in vivo and in vitro proved that the mixed micelle system composed of Solutol^®^HS15 and TPGS could not only improve the solubility of drugs, but also increase the concentration and retention time of drugs in the tumor site, and finally increase the anti-tumor effect. Thus, this system could serve as a promising carrier of Rh_2_ for cancer therapy.
